# Inactivation influences the extent of inhibition of voltage-gated Ca^+2^ channels by Gem—implications for channelopathies

**DOI:** 10.3389/fphys.2023.1155976

**Published:** 2023-08-16

**Authors:** Salma Allam, Rose Levenson-Palmer, Zuleen Chia Chang, Sukhjinder Kaur, Bryan Cernuda, Ananya Raman, Audrey Booth, Scott Dobbins, Gabrielle Suppa, Jian Yang, Zafir Buraei

**Affiliations:** ^1^ Department of Biology, Pace University, New York, NY, United States; ^2^ Department of Biological Sciences, Columbia University, New York, NY, United States

**Keywords:** ataxia, Ca^2+^, channelopathy, GTPase, heart, ion channel, inactivation, muscle

## Abstract

Voltage-gated Ca^2+^ channels (VGCC) directly control muscle contraction and neurotransmitter release, and slower processes such as cell differentiation, migration, and death. They are potently inhibited by RGK GTP-ases (Rem, Rem2, Rad, and Gem/Kir), which decrease Ca^2+^ channel membrane expression, as well as directly inhibit membrane-resident channels. The mechanisms of membrane-resident channel inhibition are difficult to study because RGK-overexpression causes complete or near complete channel inhibition. Using titrated levels of Gem expression in *Xenopus* oocytes to inhibit WT P/Q-type calcium channels by ∼50%, we show that inhibition is dependent on channel inactivation. Interestingly, fast-inactivating channels, including Familial Hemiplegic Migraine mutants, are more potently inhibited than WT channels, while slow-inactivating channels, such as those expressed with the Cavβ_2a_ auxiliary subunit, are spared. We found similar results in L-type channels, and, remarkably, Timothy Syndrome mutant channels were insensitive to Gem inhibition. Further results suggest that RGKs slow channel recovery from inactivation and further implicate RGKs as likely modulating factors in channelopathies.

## Introduction

Voltage-gated Ca^2+^ channels (VGCC) are critical for nerve, heart, and muscle function. Their opening triggers neurotransmitter release and muscle contraction, and can initiate slower processes such as cell migration, gene transcription and cell death. Not surprisingly, mutations in VGCC have been directly implicated in epilepsy, migraine, Alzheimer’s disease, blindness, pain, schizophrenia, atrial fibrillation, and several other neurological and cardiovascular disease (Saegusa et al., 2001; Bidaud et al., 2006; Doering et al., 2007; Catterall et al., 2008; Cain and Snutch, 2011; Hurley and Dexter, 2012; Bourinet et al., 2014). The immediate effect of some of these mutations, which are often in the channel’s pore-forming subunit (Ca_v_α_1_), is to alter channel inactivation, leading to an aberrant Ca^2+^ influx. For example, Timothy Syndrome is caused by a single point mutation that dramatically slows channel inactivation, affecting both nerve and cardiac muscle function—patients suffer from arrhythmias that lead to cardiac arrest by the age of four and are often diagnosed with autism spectrum disorder (Splawski et al., 2004).

**FIGURE 1:**
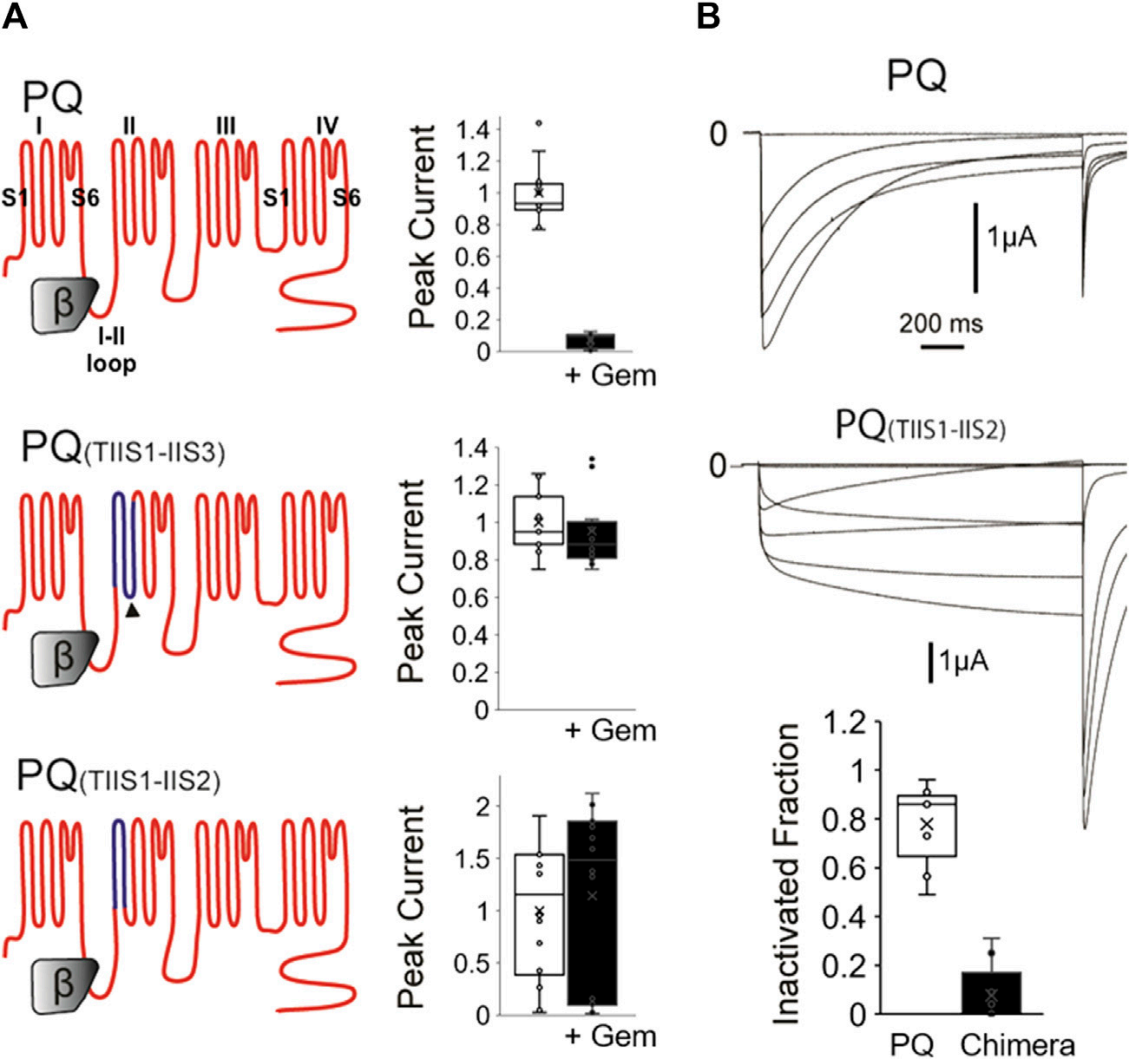
A chimeric P/Q-T channel insensitive to Gem has slow inactivation. **(A)** WT P/Q channels (top) were expressed in *Xenopus* oocytes with the β3 and α2δ subunits in the presence or absence of Gem and currents were recorded using two-electrode voltage clamp. Gem inhibits P/Q channels (top bar graph). Gem inhibition can be abolished when the IIS1-IIS3 region (dark blue topology in middle panel) is replaced with its counterpart in the Gem insensitive T-channels, which confirms a previous study (Fan et al., 2010). Interestingly, restoring the native 8 amino acid cytosolic PQ-channel region back to this chimera, which gives rise to the PQ(TIIS1-IIS2) chimera in the bottom panel, still yields Gem-insensitive channels (see methods for chimeric amino acids). **(B)** 1,500 ms long voltage pulses reveal that the PQ(TIIS1-IIS2) channel chimera is slowly inactivating compared to WT channels. Bar graph shows whisker plots of fold inactivation calculated by comparing peak current at 0 mV to leftover current at the end of the 1.5 s pulse. ** indicates *p* < 0.01.

The main pore-forming α_1_ subunit of calcium channels, Ca_v_α_1_, is composed of 4 homologous domains (I-IV, Figure 1A), each containing six transmembrane helices (S1-S6). The four domains are connected by intracellular loops named I-II, II-III, and the III-IV loop. In high voltage-activated (HVA) calcium channels, which are L-, N-, P/Q- and R-type, the I-II loop contains a region that anchors the auxiliary cytosolic β subunit, or Ca_v_β (Chen et al., 2004; 2004; Van Petegem et al., 2004). The β subunit is essential for channel trafficking to the membrane, and controls channels gating. For example, the Ca_v_β2a subunit, whose expression is developmentally regulated, dramatically slows channel inactivation (Olcese et al., 1994; Buraei and Yang, 2010).

The presence of the β subunit is also required for HVA channel regulation by the small monomeric RGK-GTPases: Rem, Rem2, Rad, Gem/Kir (Buraei and Yang, 2010). RGKs dramatically inhibit all HVA channels (L-, N-, P/Q- and R-type channels), while low voltage-activated calcium channels (LVA; CaV3, or T-type channels) are resistant to inhibition (Béguin et al., 2001; Finlin et al., 2003; Chen et al., 2005; Seu and Pitt, 2006; Yada et al., 2007; Bannister et al., 2008; Flynn et al., 2008; Fan et al., 2010). Two types of inhibition can occur: a slow, trafficking-dependent mechanism (Béguin et al., 2001; Beguin et al., 2005; Béguin et al., 2006; Fan et al., 2010), and a fast mechanism that inhibits membrane-resident channels (Chen et al., 2005; Finlin et al., 2005; Xu et al., 2010). While the inhibition of trafficking seems to be dependent on dynamin-mediated endocytosis, a number of different mechanisms have been proposed for the inhibition of membrane-resident channels, including the formation of a “non-conducting” channel pore (Chen et al., 2005), voltage sensor immobilization (Yang et al., 2010), and a great decrease in channel open probability (Seu and Pitt, 2006; Yang et al., 2010). However, one great challenge in studying RGK-mediated inhibition of membrane-resident channels, is that inhibition is very potent, leaving exceedingly small, or no currents to study (Béguin et al., 2001; Finlin et al., 2003; Chen et al., 2005; Seu and Pitt, 2006; Yada et al., 2007; Bannister et al., 2008; Flynn et al., 2008; Fan et al., 2010). Yet, physiological levels of inhibition that are uncovered *in-vivo* in RGK knockout mice, or siRNA-treated tissues reveal levels of inhibition ranging from 15%–55% (Yada et al., 2007; Wang et al., 2010; Yamakawa et al., 2014; Scamps et al., 2015). Thus, it is not clear which, if any, of these mechanisms of inhibition impact functional surface channels.

In a previous study, we rendered a P/Q channel completely insensitive to RGK inhibition, by replacing its IIS1-IIS3 region with that of the RGK- insensitive LVA T-type channel, CaV3.1 (Fan et al., 2010). It was not clear from this study, however, why this chimera, named PQ(TIIS1-IIS3), is insensitive to inhibition.

In this study, we investigated Gem inhibition of P/Q- and L-type Ca^2+^ channels (Cav2.1 and Cav1.2 respectively) expressed in *Xenopus* oocytes. Using titrated low levels of Gem expression that inhibit currents by ∼50% we revealed that Gem stabilizes P/Q channels in the inactivated state and slows recovery from inactivation. We also found that Gem differentially inhibits WT channels versus mutants with altered inactivation, including mutants associated with known channelopathies. Most notably, the Timothy Syndrome mutation renders L-type channels insensitive to Gem, exposing RGKs as potential players in this and other channelopathies.

## Results

As mentioned, we had previously created an RGK-insensitive chimeric P/Q channel harboring the IIS1-IIS3 region from T-channels. Compared to WT channels, which were significantly inhibited (Figure 1A, top), this chimera was insensitive (Figure 1A middle), confirming our previous study (Fan et al., 2010). Here, we wondered whether this chimera had become insensitive to RGKs because it lost a cytosolic-facing P/Q-channel region comprised of eight presumably cytosolic residues in the cytoplasmic linker between IIS2 and IIS3 (Figure 1A dark arrowhead) that may have served as a Gem binding site. Thus, we restored this presumed cytosolic domain in a new chimera named PQ_(TIIS1-IIS2)_ and shown at the bottom of Figure 1A. Compared to WT channels, this construct was *still* insensitive to RGK inhibition, which was puzzling, so we proceeded to biophysically interrogate this PQ chimera.

Calcium channel inactivation serves a critical role in limiting Ca^2+^ influx into the cell, and is often disrupted in calcium channel mutants and calcium channelopathies. Thus, to investigate whether the chimeras had unique biophysical properties that interfered with Gem inhibition, we first examined channel inactivation. We coexpressed WT PQ or chimeric PQ_(TIIS1-IIS2)_ channels with β3 and α2δ subunits in *Xenopus* oocytes and studied their inactivation properties using 1,500 ms long voltage steps, instead of the usual 50 ms steps. Interestingly, we found that chimeric channels inactivated at a dramatically reduced rate compared to WT channels when stimulated with long voltage pulses (Figure 1B, 7% ± 4% vs. 77% ± 5% inactivation at 0 mV for mutant vs. WT channels respectively, *p* < 0.01).

We thus wondered whether slowed channel inactivation *per se* was causing the chimera’s resistance to RGK inhibition. If there was a causative relationship between the two, i.e., if slowed channel inactivation underlies the insensitivity to RGKs, then any mutation outside of the IIS1-IIS2 region that slows inactivation should have a similarly protective effect. Furthermore, if RGK-mediated inhibition was indeed dependent on channel inactivation, then mutations that speed channel inactivation should heighten RGK inhibition.

**FIGURE 2:**
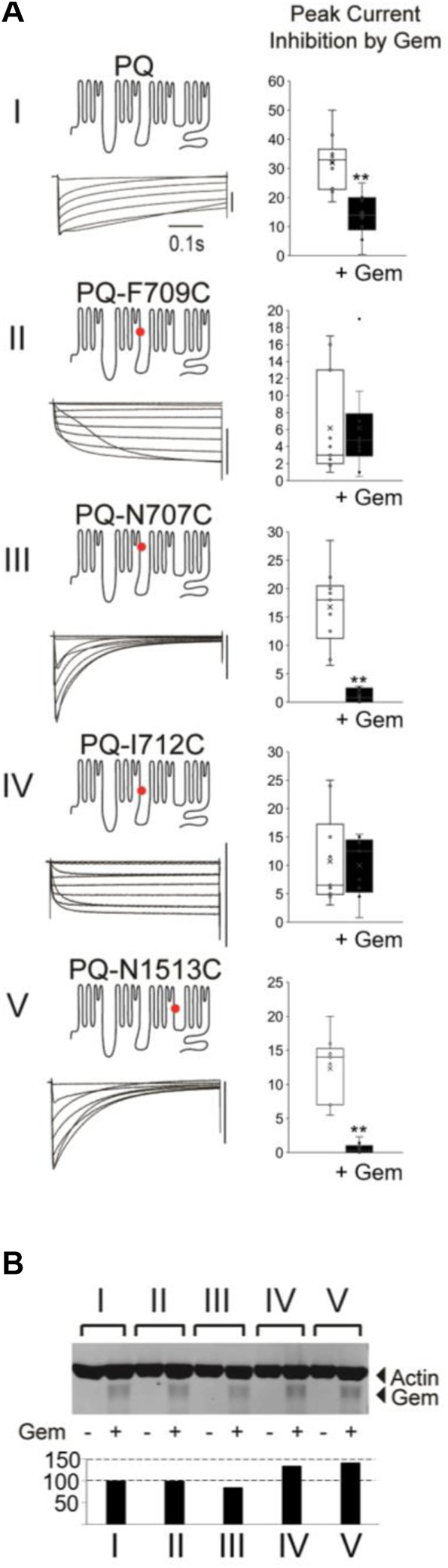
Different mutations in PQ channels that alter inactivation also alter sensitivity to Gem. **(A)** Various mutant channels (red dot indicates mutation site) with differing inactivation kinetics (see Supplementary Figure S1) were expressed in *Xenopus* oocytes and their currents during 0.5 s voltage steps recorded. The vertical bar to the left of the currents indicates 10 µA. Currents were recorded in the presence or absence of Gem but shown here are those in the presence or absence of HA-Gem. In this set of experiments the amount of HA-tagged Gem RNA injected into the oocytes was adjusted to a level that gives ∼50% inhibition in WT P/Q-channels (top bar gaph) so both increases and a decreases in Gem potency can be observed in the mutants. Ten Oocytes were collected after recording for each of the experimental groups in the westerns in **(B)** which show similar amounts for HA-Gem expression for all conditions. See *Methods* for HA-Gem quantification.

To that end, we introduced several single point mutations into different regions of the P/Q channel, producing channels with very different inactivation properties—mutations F709C and I712C slowed inactivation, while N707C and N1513C significantly sped the time constant of inactivation, three-fold, compared to WT channels (Figure 2A; Supplementary Figure S1). To ensure that there are sufficient currents to study, we injected a concentration of Gem cRNA that produces ∼50% inhibition of WT P/Q-channels [see Methods (Canti et al., 2001; Seu and Pitt, 2006)]. Finally, to ensure that differences in the magnitude of Gem inhibition between different channels are not due to different levels of Gem expression, we repeated all of the experiments with HA-Gem, which we had previously shown to be equally functional and as potent as WT Gem (Fan et al., 2010; Fan et al., 2012). Gem and HA-Gem yielded similar levels of inhibition, of which the HA-Gem results are shown below (Figure 2; the results with untagged Gem are in Supplementary Figure S2). To our surprise, mutant channels with slowed inactivation were not inhibited. On the other hand, mutant channels with fast inactivation kinetics were inhibited by Gem and HA-Gem, more so than WT channels (Figure 2; Supplementary Figure S2). To test whether the observed differences in channel inhibition resulted from large differences in HA-Gem expression, we analyzed the very same oocytes from which recordings were obtained using western blots for levels of HA-Gem expression, which were found to be similar in all conditions (Figure 2B).

These results suggest the possibility that channel inactivation, independent from a particular channel region, can dictate the strength of Gem inhibition.

### The β2a subunit ameliorates Gem-mediated inhibition

**FIGURE 3:**
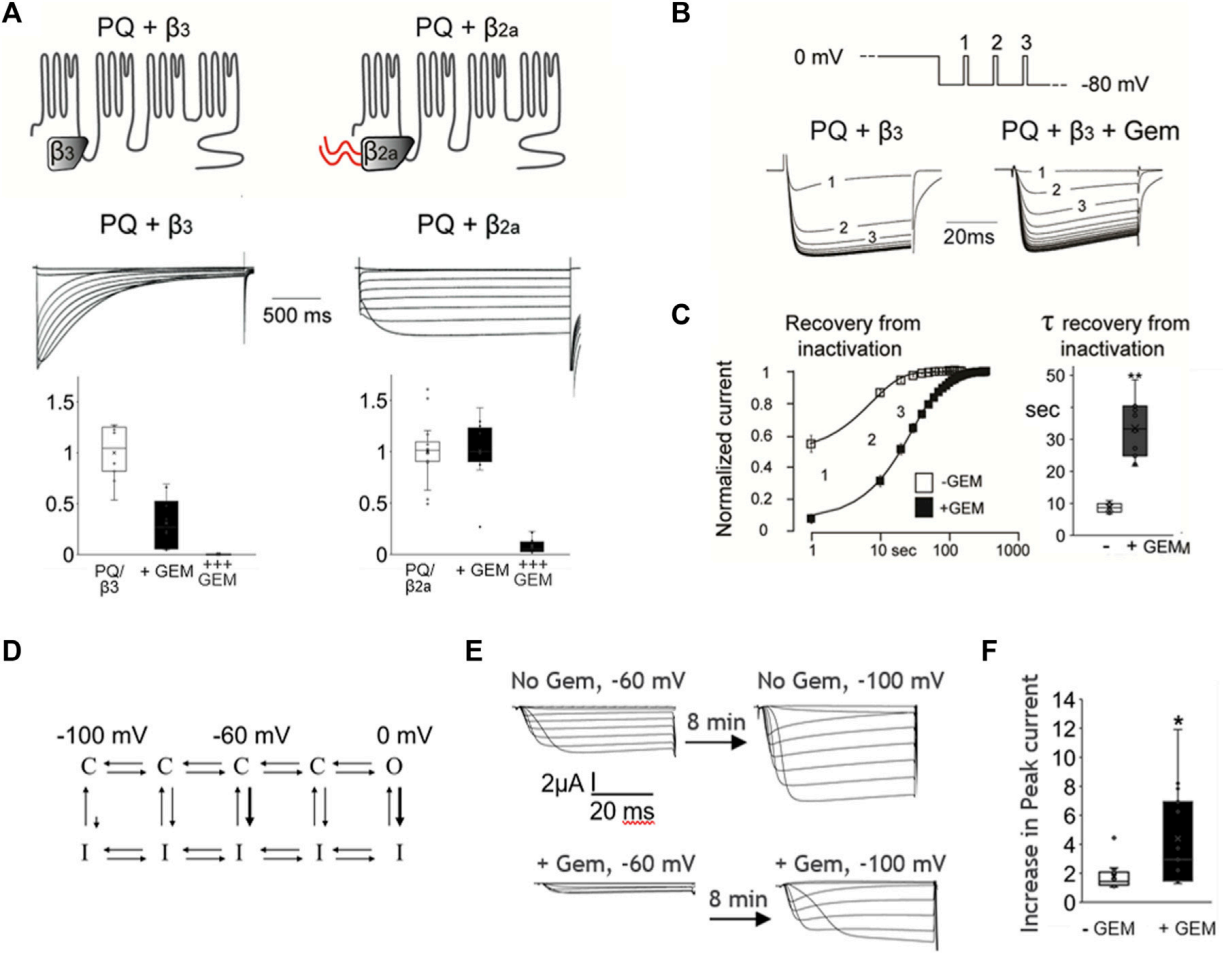
Coexpression of the Ca_v_β_2a_ subunit can abolish Gem inhibition of P/Q-channels and Gem delays channel recovery from inactivation. **(A)** Co-expression of P/Q channels with the β2a subunit, which slows inactivation, abolishes inhibition by Gem (“+Gem” indicates the addition of 0.03 ng/cell of Gem cRNA). Inhibition can be restored using larger amounts of injected Gem cRNA (“+++” bar graphs are for cells with with 0.3 ng of Gem cRNA/cell). **(B)** Recovery from inactivation was studied using minute long pulses to 0 mV followed by recovery from inactivation at a holding potential of −80 mV. During recovery, short test pulses to 0 mV were given every 10 s (top panel) and currents recorded in the presence or absence of Gem (lower panel). **(C)** The average time course of recovery from inactivation (note log scale); currents were normalized to post-recovery levels. Smooth lines show an exponential fit to the average time course of recovery (τ = 8.6 s without Gem and 34.3 s with Gem). Bar graphs show standard whisker plots for the time constants, which were significantly different, *p* < 0.01. **(D)** A diagram based on Bähring and Covarrubias (2011) helping visualize transitions between closed (C), open (O), and inactivated (I) states. See text. **(E)** Cells without Gem (upper current panels) or with Gem cRNA (lower current panels), alongside cRNAs for the PQ channel (Cav2.1) pore-forming subunit cRNA, β3, and α2δ subunits were held at −60 mV for 8 min, their currents recorded (left side currents), then switched to a holding potential of −100 mV for 8–10 min, and their currents recorded again (right side currents). The increase in current size was expressed in **(F)** as a fold increase in current in −100 mV compared to −60 mV.

To further probe this hypothesis we took advantage of the fact that HVA channels can be modulated with a unique β subunit splice variant, β2a, that dramatically slows channel inactivation [Olcese et al., 1994; reviewed in Buraei and Yang (2010)]. We thus coexpressed WT P/Q channels with either β2a or the β3 subunit (which does not slow inactivation) and compared their inhibition by Gem. As shown in Figure 3, β2a-containing channels inactivated slowly, as expected [Olcese et al., 1994; reviewed in Buraei and Yang (2010)], and we found them to be insensitive to Gem compared to channels with the β3 subunit. These results were somewhat surprising because previous reports indicated complete channel inhibition by RGK proteins in the presence of the β2a subunit (Finlin et al., 2003; Yang et al., 2010; 2012), albeit, in a HEK cell over-expression system. We wondered whether this discrepancy between earlier and our results stems from differing levels of Gem expression and tested this idea by increasing the concentration of injected Gem cRNA from 0.03 ng/cell to 0.3 ng/cell. Under these conditions, we were able to replicate the previously reported strong inhibition of currents, and the inhibition of β2a-paired channels was nearly complete (Figure 3A; +++ Gem).

### Gem slows channel recovery from inactivation

One interpretation of the results thus far is that channels that rarely enter the inactivated state are less sensitive to Gem while those that readily enter the inactivated state are more sensitive to Gem. A possible mechanism for this could be that Gem stabilizes channels in the inactivated state. If this is true, Gem should slow channel recovery from inactivation. To investigate this idea, we studied P/Q channel recovery from inactivation in the presence or absence of Gem; we used brief pulsing to assess current recovery following a long, 2 s inactivating pulse to 0 mV. As shown in Figures 3B, C, recovery from inactivation in the absence of Gem was fast, with a time constant of ∼8.6 s. On the other hand, in the presence of Gem, channels recovered from inactivation significantly slower, with a time constant of ∼33.4 s, suggesting that Gem may retard channel recovery from inactivation (*p* < 0.01).

The pulse to 0 mV promotes inactivation from the open state (Figure 3D, thick arrow near 0 mV), but VGCC can also inactivate from “intermediate” closed states (Zhang et al., 1994; Patil et al., 1998; Yasuda et al., 2004; Bähring and Covarrubias, 2011; Sheng et al., 2012), e.g., when the cell is at −60 mV, as compared to the limited inactivation from “deep” closed states that are far from the open state, e.g., when the cell is at −100 mV (Figure 3D, thick arrow near −60 mV, and small arrow near −100 mV). To investigate whether Gem slows recovery from closed-state inactivation, we wanted to study steady state inactivation, but steady state took 5–8 min to reach in the presence of Gem. So instead, we compared currents elicited from resting potentials during which channels are expected to be closed, waiting 8–10 min at each, before measuring peak currents (Figure 3E), similar to (Yasuda et al., 2004)); we used −60 mV and −100 mV. In the absence of Gem, and with some channels locked in the inactivated state at −60 mV, we found that cells recovered 1.77 fold more current when transferred to a holding potential of −100 mV. However, in the presence of Gem, cells recovered 4.4-fold more current upon switching from −60 mV to −100 mV (Figure 3F, *p* < 0.05), suggesting that a larger proportion of channels at −60 mV is in the closed inactivated state. This was reversible and the switch from a holding potential of −100 mV to −60 mV inactivated more channels in the presence of Gem (36% ± 17 inactivation with Gem versus 15% ± 13 without Gem; *p* < 0.01, Supplementary Figure S2). Future experiments will investigate biophysical properties that will help determine the different state transition rates and help model Gem’s action, but the current results indicate that Gem likely stabilizes one or more inactivated states.

### Gem differentially inhibits WT channels versus those associated with channelopathies

We next wondered whether Gem inhibition could be a contributing factor in channelopathies, many of which are associated with altered inactivation properties. One of the most studied calcium channelopathies, Timothy Syndrome (TS), is an L-type Ca_v_1.2 channelopathy characterized by arrhythmias and, often, autism or autism spectrum disorder (Splawski et al., 2004). Different mutations can cause TS; most of which are similarly malpositioned at the very proximal end of the I-II loop, where a glycine residue is thought to act as a the flexible hinge for a hinged-lid mechanism that inactivates the channel (Stotz et al., 2000). Thus, the G406R mutation, which can be found in either one of the alternatively spliced exons 8 and 8a, and the G402S mutation in exon 8 (Splawski et al., 2004; 2005; Fröhler et al., 2014), all severely reduce channel inactivation (Splawski et al., 2004; Barrett and Tsien, 2008). Previous studies showed normal inhibition of TS channels by RGKs (Krey and Dolmetsch, 2009; Krey et al., 2013).

**FIGURE 4:**
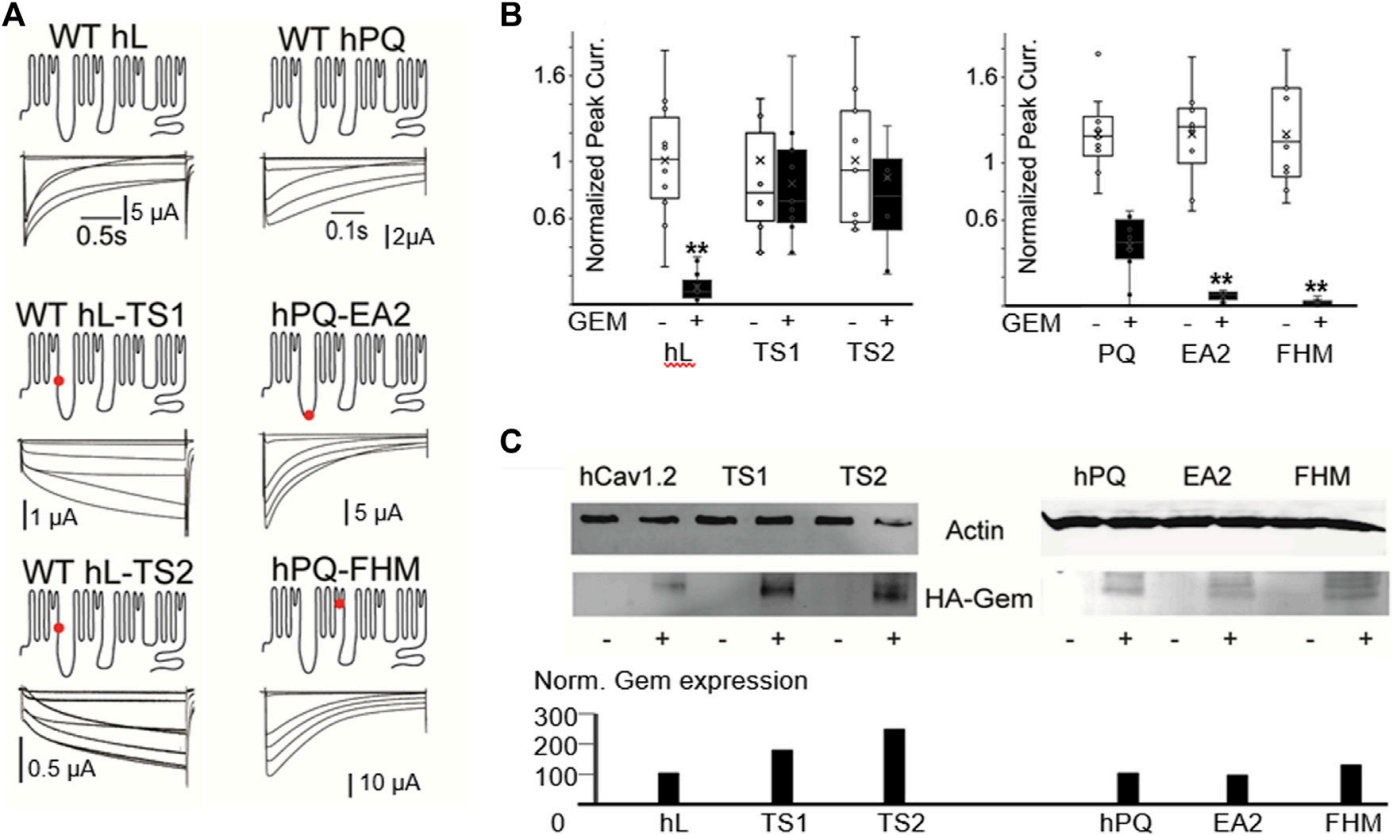
Human disease mutations dramatically alter sensitivity to Gem inhibition. **(A)** WT and the indicated disease mutants of human PQ and L-channels were coexpressed with β3 and α2δ subunits. Red dots indicate mutations sites. **(B)** T. S. mutants show dramatic resistance to Gem inhibition, while PQ channel mutants exhibit increased inhibition. White whisker plots are normalized peak currents from cells without HA-Gem, while black bars reflect fold reduction in peak currents from cells co-expresing HA-Gem. Differences in average current amplitudes were statistically significant at the *p* < 0.01 level, as indicated by the “**”. Similar results with untagged Gem are in Supplementary Figure S4. **(C)** Westerns show HA-Gem levels in cell lysates (pooled from 8 oocytes for each condition), and were quantified using ImageJ. N ≥ 8 for each sample.

To test whether Gem could differentially regulate WT and TS channels, and to simultaneously test whether our findings in P/Q channels, which belong to the CaV2 family of VGCC extend to L-type channels, we introduced the above two mutations that can cause Timothy Syndrome into human Cav1.2 (L-type) channels. The mutations dramatically slowed channel inactivation (Figure 4A), as expected (Splawski et al., 2004). Surprisingly, both TS mutants exhibited significant resistance to Gem inhibition compared to WT L-channels (with HA-Gem, 88% inhibition of WT channels *versus* a 16% and 12% inhibition for mutant L-channels; *p* < 0.01; Figure 4B; see similar results with untagged Gem in Supplementary Figure S4). This was the case in spite of similar, or slightly higher levels of HA-Gem expression with the mutants (Figure 4C).

Next, we investigated two point mutations in the PQ-type Ca^2+^ channel that speed channel inactivation: one associated with Familial Hemiplaegic Migraine, with a devastating type of progressive cerebellar atrophy, ataxia, and, often, a coma [FHM; T666M located in the pore loop of domain II (Hans et al., 1999; Choi et al., 2012; Li et al., 2019)]; and the other PQ channel mutation is associated with Episodic Ataxia 2 [EA2; A454T located in the distal region of the I-II loop (Cricchi et al., 2007; Serra et al., 2009)]. These mutations speed channel inactivation, and we hypothesized they would be prone to a stronger Gem-mediated inhibition. To test this, we used the human P/Q channel to introduce mutations associated with FHM or EA2 (Figure 4). As expected, these mutations sped inactivation ∼3 fold (Supplementary Figure S1), and, as hypothesized, these mutants were *hypersensitive* to RGK inhibition and were significantly more inhibited compared to WT channels (62% inhibition of WT versus 94% and 98% inhibition for FHM and EA2 respectively, *p* < 0.01 for both, Figures 4B, C; Supplementary Figure S4 shows similar results with untagged Gem).

## Discussion

In this paper, we demonstrate several important findings. 1) Gem inhibition of P/Q-channels in expression systems is not necessarily complete, and can be titrated in *Xenopus* oocytes to achieve a lower level of inhibition that is more physiologically relevant. 2) Gem slows P/Q-channel recovery from inactivation, and has a differential effect on channels with differing inactivation kinetics. 3) The β_2a_ subunit of VGCC, which slows inactivation, blunts RGK-mediated inhibition. 4) Timothy Syndrome channels are insensitive to Gem inhibition, suggesting they lack a key aspect of regulation that would have potentially curbed the excessive Ca^2+^ influx into cells. In addition, Gem is a likely exacerbating factor in several human disease mutations that alter calcium channel inactivation.

Most studies of RGK proteins are overexpression studies in HEK293 cells, or other systems, where VGCC currents are nearly completely abolished, complicating mechanistic studies (Béguin et al., 2001; Finlin et al., 2003; Chen et al., 2005; Seu and Pitt, 2006; Yada et al., 2007; Bannister et al., 2008; Flynn et al., 2008; Fan et al., 2010; Yang et al., 2010; 2012), and raising doubts as to whether RGK proteins naturally inhibit VGCC considering they have other critical functions related to cytoskeletal reorganization and dendritic arborization (Ghiretti and Paradis, 2011; 2014). However, numerous *in vivo* and *in situ* RNAi knockdown, knockout, and dominant negative studies reported levels of inhibition ranging from 30% to 50% in various tissues, primary cell cultures, and cell lines (Yada et al., 2007; Wang et al., 2010; 2011; Manning et al., 2013; Scamps et al., 2015) and multiple recent studies have implicated RGKs in critical physiological functions related to VGCC inhibition (Scamps et al., 2015; Yamakawa et al., 2014; reviewed in Buraei and Yang, 2015; Miranda et al., 2021; 2021).

Here, we took advantage of two previous studies establishing that *Xenopus* oocytes can be used to carefully control the levels of calcium channel subunit expression (Canti et al., 1999) and that RGKs can inhibit L-channels in a manner dependent on the dose of the injected RGK cRNA (Seu and Pitt, 2006), and we titrated Gem levels to achieve ∼50% channel inhibition to reveal a novel form of Gem-mediated channel regulation where Gem slowed P/Q channel recovery from inactivation (Figure 3). One direct consequence we found is that channels that more readily inactivate are also more readily inhibited by Gem, while those that are more resistant to inactivation are, too, resistant to Gem-mediated inhibition. Physiologically, this may fine-tune processes that critically depend on VGCC inactivation such as neurotransmitter and hormone release, neural excitability, cardiac and skeletal muscle contraction, and other processes, all of which were shown could be modulated by Gem (Xu et al., 2014; Scamps et al., 2015; Meza et al., 2018; Miranda et al., 2021). For example, Gem was shown to be upregulated during nerve injury to reduce PQ channel activity and likely reduce regenerating neurite branching during regeneration (Wang et al., 2013; Scamps et al., 2015), and Gem-mediated inhibition of L-type channels was shown to control light-induced phase shifts in circadian rhythms in the suprachiasmatic nucleus (Matsuo et al., 2022).

Our finding that Gem differentially reduces the fraction of available channels in intermediate closed states (at −60 mV, Figure 3) has implications for cells with high frequency action potentials that push channels into the intermediate closed states, as first described for VGCC by Patil et al. (1998). Under those circumstances, Gem is expected to reduce the pool of available channels and to limit excitability and potential cytototoxicity.

One of the most potent physiological regulators of calcium channel gating and particularly channel inactivation is the Cavβ subunit, whose tissue and developmental regulation is tightly regulated (Buraei and Yang, 2010). The Cavβ_2a_ subunit is a unique splice variant the dramatically slows inactivation of any partnered high-voltage activated Ca^2+^ channel. Interestingly, our results suggest that β_2a_ can disengage channels from Gem inhibition (Figure 3). It is noteworthy that several previous studies reported complete channel inhibition by Rem and Rad in the presence of β_2a_ (e.g., Finlin et al., 2003; Yang et al., 2010). It could be that inhibition by Gem is mechanistically different, but a more likely explanation is that Rem and Rad were overexpressed, which may have masked the effect of β_2a_ to counter RGK inhibition. It would be interesting to investigate how critical is the interplay between Gem inhibition and β_2a_ subunit expression in the function of brain, heart and aorta, where β_2a_ is predominantly expressed. A recent outstanding study of mutant β_2_ subunits implicated in autism spectrum disorder demonstrated differences in Gem-mediated inhibition of single L-type channels partnered with different mutant β_2_ subunits. Whole cell currents were difficult to analyze in this study (due to the strong Gem-mediated inhibition) and it was not clear whether Gem expression levels were similar in HEK cells, but single channel recordings beautifully demonstrated a differential effect imparted by the different β_2_ subunit mutants on Gem-mediated changes in single channel properties (Despang et al., 2020). This further underscores Gem as an important player in calcium channelopathies.

RGK proteins have been previously implicated in other human disease. For example, the failing heart has a three-fold higher level of Gem (Tan et al., 2002) and a donwregulated level of Rad (Chang et al., 2007); Gem and Rem2 have both been implicated in deficient insulin secretion from pancreatic β cells (Finlin et al., 2005; Gunton et al., 2012). In this study, we found that several mutant channels associated with neurological and cardiovascular disorders are inhibited by Gem to a significantly different extent compared to WT channels. The Familial Hemiplegic Migraine autosomal dominant mutation T666M is the most frequent cause of FHM1 and has been identified in many studies to be associated with hemiparesis, cerebellar atrophy, progressive cerebellar ataxia, coma, hallucinations, and, in several families, with intellectual disability and other cognitive impairments (Kors et al., 2003; Freilinger et al., 2008; Yabe et al., 2008; Karner et al., 2010; Choi et al., 2012; Li et al., 2019). One study found that currents are severely reduced by this mutation but the mechanism was unclear and it did not seem to result from a reduced channel surface expression (Tao et al., 2012). A different study investigating T666M channel properties found an increased channel inactivation and reduced recovery from inactivation (Kraus et al., 1998). Our studies of this devastating mutation, whose symptoms can appear in childhood (Ohmura et al., 2012), indicate that an increase in Gem-mediated inhibition could be the principal culprit. This identifies Gem as an excellent target for potential FHM1 treatment.

Episodic Ataxia 2 (Cricchi et al., 2007; Serra et al., 2010) is a rare disorder with an early onset characterized by progressive ataxia and one of the P/Q-channel mutations associated with EA2 is A454T, which was shown to have stronger inactivation coupled with a reduced modulation by Syntaxin 1A and SNAP-25, which reduced exocytosis (Serra et al., 2010). Our study suggests that, in contrast to the impaired modulation by Syntaxin and SNAP-25, Gem-mediated inhibition is increased, again highlighting Gem as a potential target for treatment. Other VGCC modulators have been found that differentially impact WT versus channels implicated in disease. For example, G-proteins differentially inhibit WT versus some mutant PQ-channels that cause Familial hemiplegic migraine (Garza-López et al., 2012). Nevertheless, Gem is one of the strongest known regulators of VGCC (Buraei and Yang, 2015) and, as such, may be a very effective target to study.

Finally, our study reveals a dramatically reduced effectiveness of Gem to inhibit Timothy Syndrome channels, Importantly, based on our westerns here and elsewhere in the paper, the levels of Gem expression did not predict the levels of inhibition. For example, the highest levels of HA-Gem expression was in oocytes coexpressing the TS2 mutation (G402S), yet they were not inhibited (Figure 4).

While rare, the slowed inactivation in TS fatally prolongs the QT interval. Autism, on the other hand, is diagnosed only in a subset of patients (Cheng et al., 2011; Dixon et al., 2012; Krey et al., 2013). The differences between patient’s who have the same (L-type channel) mutation, are likely due to compensatory mechanisms by other proteins, some of which may interact with and modulate calcium channels, such as RGK proteins. Recently, a role for Gem in the etiology of Timothy Syndrome was highlighted when TS channels were found to interact 50% less effectively with Gem compared to WT channels. This was thought to lead to altered dendritic morphology and arborization in TS neurons (Krey et al., 2013). However, in contrast to the findings in our study, where TS channels were resistant to inhibition, TS channel inhibition by Gem appeared normal (Krey and Dolmetsch, 2009; Krey et al., 2013). The difference in the two studies is likely due to the difference in the levels of Gem expression. To the best of our knowledge, this is the first report showing resistance to RGK-mediated inhibition of Timothy Syndrome channels.

Interestingly, several other proteins differentially modulate WT vs. TS channels. To give two examples, CamKII phosphorylates TS channels at a novel consensus site created by the mutation, which may contribute to the channels’ slowed inactivation (Erxleben et al., 2006; Thiel et al., 2008), since pharmacological inhibition of CamKII restores inactivation and normalizes gating (Erxleben et al., 2006; Thiel et al., 2008). Also, AKAP150 is thought to be abnormally coupled to TS, but not WT channels, and AKAP150 ablation normalized currents (Cheng et al., 2011). It would be interesting to investigate Gem-mediated inhibition as a potential ‘rescue’ for TS channels.

Recently, great potential has been revealed for the use of RGK proteins in gene therapy for calcium channel-related cardiovascular disease (Murata et al., 2004; Yang et al., 2007). Our results suggest they are also exacerbating factors in several neurological and cardiovascular disease. This makes their exploration as possible therapeutic targets very timely.

## Materials and methods

### Oocyte preparation and expression

Ovarian lobes were obtained from adult *Xenopus laevis* (from *Xenopus* One) under anesthesia in a manner approved by the institutional IACUC, and some were obtained directly from *Xenopus* one. Stages V–VI oocytes were prepared by treatment with 2.5 mg/mL collagenase A (Boehringer Mannheim) for 1.5–2.5 h in a shaking incubator at RT and 200 rpm, in a solution containing 82.4 mM NaCl, 2.5 mM KCl, 1 mM MgCl2, and 5 mM hepes (pH 7.6). Next, they were rinsed twice (15 min each time) with ND96 solution containing 96 mM NaCl, 2.5 mM KCl, 1 mM MgCl2, 5 mM hepes, 1.8 mM CaCl2, 100 units/mL penicillin, and 100 μg/mL streptomycin (pH 7.6). Single defoliculated oocytes were individually selected under a dissection scope and 50 nL cRNA mixtures injected using a Nanoject II from Drummond. The cRNA were synthesized and capped *in vitro*, and varying amounts (0.03–5 ng) were injected into selected oocytes in various combinations, and the oocytes incubated at 18°C for 3–5 days before recordings. The cDNAs encoding various constructs were subcloned into a modified oocytes expression vector pGEMHE. The constructs included wildtype (WT) or mutated human Cav2.1 (isoform 2), WT or mutant human cardiac Cav1.2, rat skeletal muscle α2δ, WT rat brain β3, WT human skeletal muscle Gem or N-terminally HA tagged Gem, and human Cavβ2a. For the PQ_(TIIS1-IIS2)_ channel chimera, PCR mutagenesis was used and CaV2.1 (GenBank accession number X57477) residues R482-G542, comprising IIS1-IIS2, were replaced with residues K738-G798 from Cav3.1 (AJ012569). In the IIS1-IIS3 chimera, CaV2.1 residues 482-K572, were replaced with residues K738-G828 of CaV3.1, as we previously described (Fan et al., 2010). The IIS2-IIS3 presumably cytosolic linker in CaV2.1 is comprised of residues T543-S550 (TRPYFHSS), while that in CaV3.1 is comprised of P799-P806 (PFGYIKNP).

### Electrophysiology

All experiments were performed at 22°C. Whole-oocyte recordings used two-electrode voltage clamp (OC725 from Warner Instruments), electrodes were filled with 3 mM KCl and had a resistance of 0.5–1 MΩ. The bath solution contained (in mM) 40 Ba(OH)2, 50 NaOH, 2 KCl, 2 BaCl2, and 5 hepes; pH was adjusted to 7.4 using methanesulfonic acid, and the solution was filtered to remove impurities. All data were analyzed with Clampfit and were represented as mean ± SD (N = number of observations). Significance was determined using two-tailed Student’s t-test. Voltage protocols were from a holding potential of −80 mV and 50 ms or 1,500 ms voltage steps ranging from −60 to +50 were applied in increments of 10 mV every 1 or every 10 s (for the longer steps).

### Western blots

The oocytes recorded using TEVC were frozen in liquid nitrogen immediately after usage. Cell lysis was performed by homogenization of 10 oocytes using a 25-G needle in a ∼15–20 µL/oocyte of a PBS solution supplemented with 1 mM EDTA, 10% Glycerol, 1%Triton, and 1:50 protease inhibitor (Halt cocktail, Pierce). Samples were centrifuged for 30 min at 10,000 g and 25 µL of the supernatant collected and mixed with 12.5 µL 3xSDS and boiled for 10 min as a whole protein control. The rest of the supernantant was incubated with gentle mixing overnight at 4°C with 20 µL of anti-HA coated beeds (Sigma), centrifuged, washed with the lysis solution with 1% total detergent three times for 5 min followed by a 5 min incubation and elution with HA peptide (0.8 mg/mL final, Genscript) dissolved in ∼40 µL wash buffer (about 2 µL/oocyte) with 0% detergent. Following centrifugation samples we boiled and are used for gels and western blots. After electrophoresis, the protein gel was transferred to the PVDF membrane and processed with the Odyssey Western blot kit (Li-Cor). The monoclonal mouse anti-HAantibody HA.11 (Covance) was used as the primary antibody. Alexa Fluor 680 goat anti-mouse IgG (Invitrogen) was used as the secondary antibody. Images were scanned and analyzed with the Odyssey Infrared Imaging System (Li-COR). To quantify western blots, we used ImageJ and measured Gem expression in relation to the levels of actin expression in the same lane (the loading control), and expressed this value as a percentage of the Gem co-expressed with the WT channel.

## Data Availability

The datasets presented in this study can be found in online repositories. The names of the repository/repositories and accession number(s) can be found in the article/Supplementary Material.
