# Supplementary material for: Inactivation influences the extent of inhibition of voltage-gated Ca+2 channels by Gem—implications for channelopathies
Source: Front Physiol. 2023 Aug 16;14:1155976. doi: 10.3389/fphys.2023.1155976 (PMC10466392; doi:10.3389/fphys.2023.1155976)
Supplement: Supplementary file 1 [file DataSheet1.pdf]

SUPPLEMENTAL DATA:

Inactivation influences the extent of inhibition of voltage-gated Ca^{+2} channels by Gem – implications for channelopathies

Salma Allam[1†], Rose Levenson-Palmer[2†], Zuleen Chia Chang[1], Sukhjinder Kaur[1], Bryan Cernuda[1], Ananya Raman[1], Audrey Booth[1], Scott Dobbins[2], Gabrielle Suppa[1], Jian Yang[2*], and Zafir Buraei[1*]

[†] These authors contributed equally

[*] Corresponding authors: jy160@columbia.edu; zburaei@pace.edu

Supplemental Figure 1

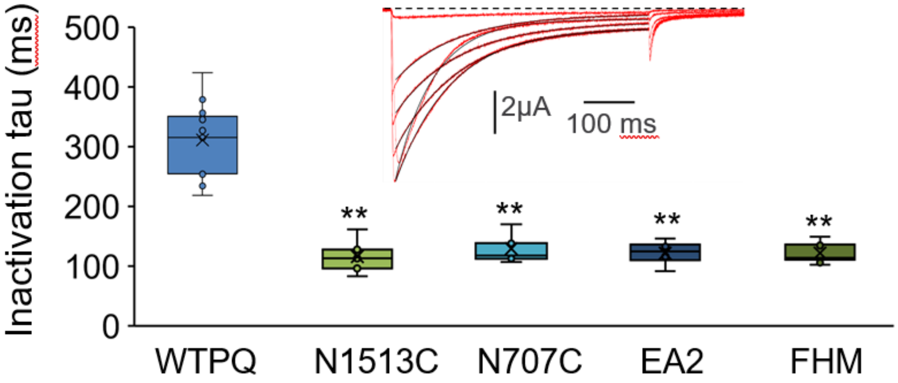

Supplementary Figure 1. Kinetics of inactivation for the indicated WT PQ channel (Cav2.1) or its fast-inactivating mutants N1513C, N707C, EA2, and FHM, show significantly reduced inactivation time constants (τ) ranging from 116 ± 30 ms for N1513C to 129 ± 26 ms for N707C, compared to WT PQ channels that have a τ of 311 ± 65 ms at peak current; $p<0.01$ for all mutants as compared to WT, n= 5-12. The inset shows how time constants of inactivation were obtained from single exponential fits (black lines) to EA2 currents (red) elicited by 500 ms long depolarizing pulses to various voltages.

Supplemental Figure 2

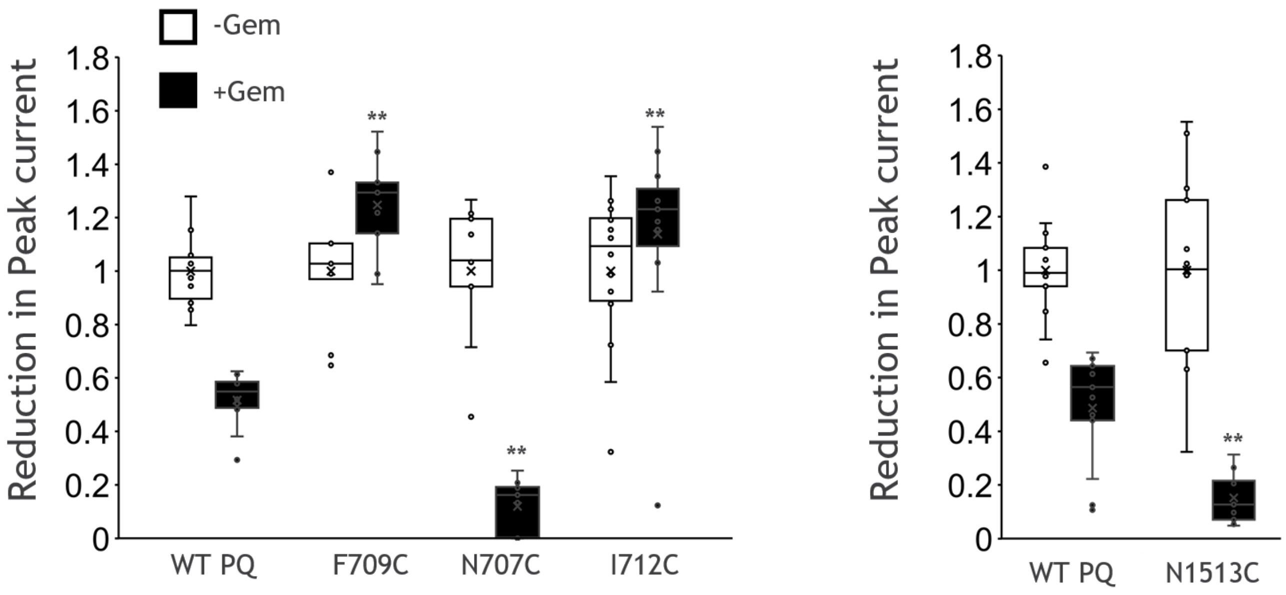

Supplemental Figure 2. Fast inactivating channels are inhibited, while non-inactivating channels are spared Gem inhibition. Oocytes were injected with the PQ channel (Cav2.1) pore-forming subunit cRNA, alongside ß3 and α2δ cRNA and either water (open bars) or Gem (black bars). Bars represent standard whisker plots; n=6-12. All differences are statistically significant from WT, at $p<0.01$.

Supplemental Figure 3

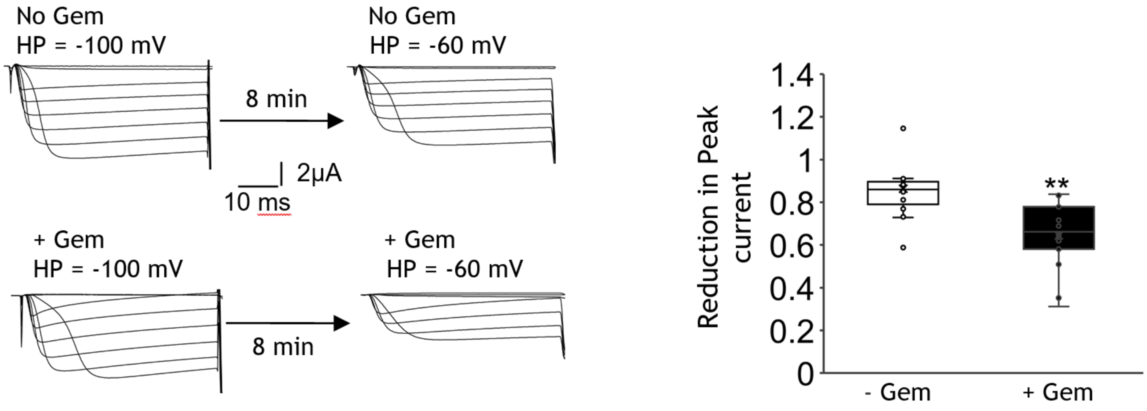

Supplemental figure 3. Cells with or without Gem, alongside the PQ channel (Cav2.1) pore-forming subunit cRNA, and the auxiliary subunits β3, and α2δ cRNA, were held at -100 mV for 8 minutes, their currents recorded (left side currents), then switched to a holding potential of -60 mV for 8-10 minutes, and their currents recorded again (right side currents). The decrease in current size was expressed as a fraction of the current at the -100 mV holding potential. In spite of the small difference, the difference is highly significant, 36% ± 17 reduction with Gem (black bar) versus 15% ± 13 without Gem; $p<0.001$.

Supplemental Figure 4

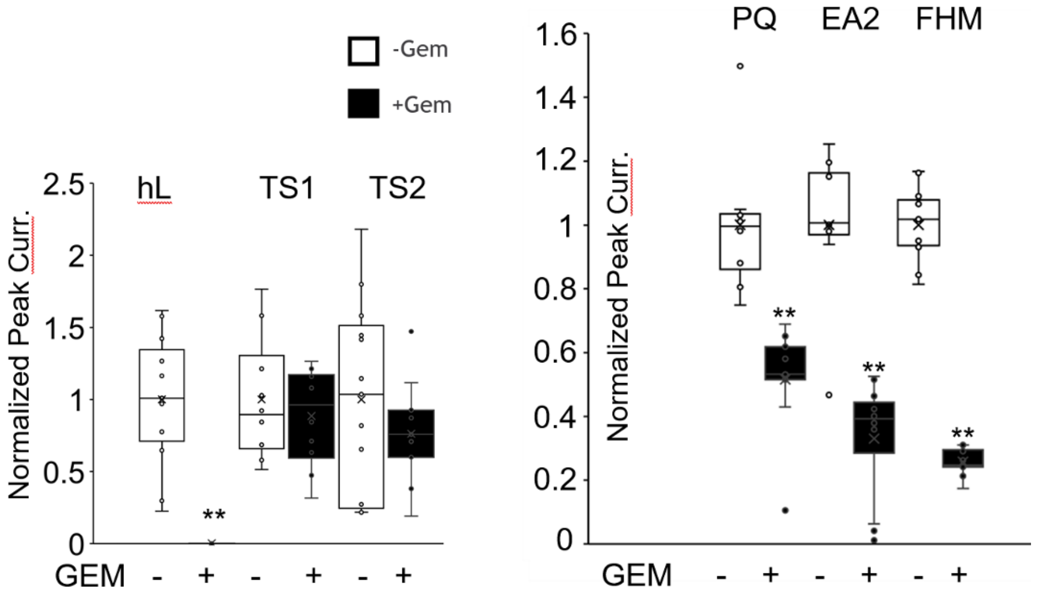

Supplemental Figure 4. Gem, co-expressed with human L-type WT or mutant channels (left graph) does not inhibit the non-inactivating Timothy Syndrome mutants as compared to WT L-channels. On the other hand, in the panel on the right, Gem inhibits fast inactivating human PQ-type channel mutants associated with Episodic Ataxia 2 (EA2) and Familial Hemiplegic Migraine (FHM) to a larger extent as compared to WT channels. P<0.01 where indicated by '**' and n = 8-10. Data in this figure are similar to those in Figure 4 but with Gem instead of HA-Gem.

Supplemental Figure 5

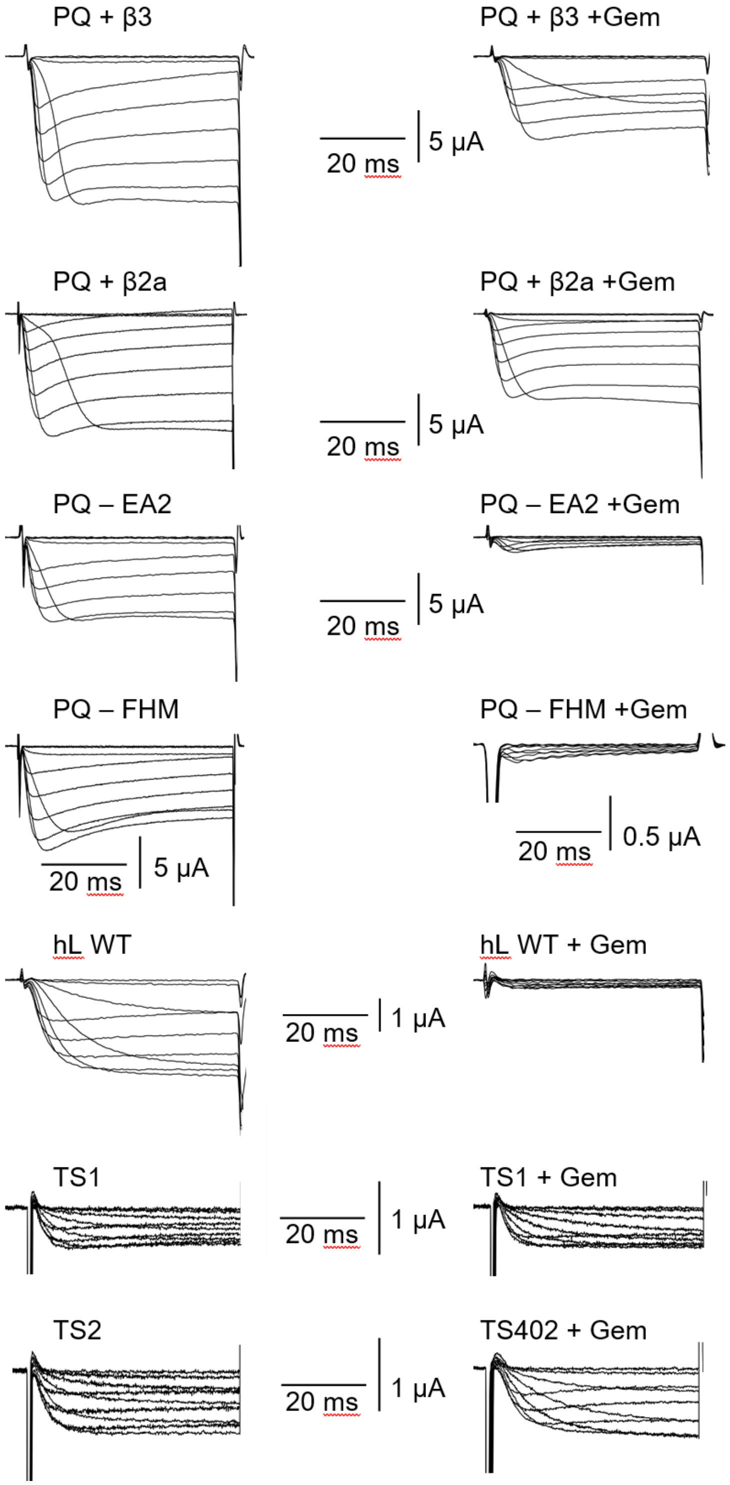

Supplemental Figure 5. Representative currents from oocytes injected with ß3 and α2δ in addition to the cRNA indicated above each panel. Each panel is a different oocyte, shown here as representative traces for current size measurements indicated in figures 3 and 4 of the main paper. Group data are in the main text. See methods for details.
